# Anthropogenically driven environmental changes shift the ecological dynamics of hemorrhagic fever with renal syndrome

**DOI:** 10.1371/journal.ppat.1006198

**Published:** 2017-01-31

**Authors:** Huaiyu Tian, Pengbo Yu, Ottar N. Bjørnstad, Bernard Cazelles, Jing Yang, Hua Tan, Shanqian Huang, Yujun Cui, Lu Dong, Chaofeng Ma, Changan Ma, Sen Zhou, Marko Laine, Xiaoxu Wu, Yanyun Zhang, Jingjun Wang, Ruifu Yang, Nils Chr. Stenseth, Bing Xu

**Affiliations:** 1 State Key Laboratory of Remote Sensing Science, College of Global Change and Earth System Science, Beijing Normal University, Beijing, China; 2 Shaanxi Provincial Centre for Disease Control and Prevention, Xi’an, Shaanxi, China; 3 Center for Infectious Disease Dynamics, Pennsylvania State University, State College, Pennsylvania; 4 Ecologie & Evolution, UMR 7625, UPMC-ENS, Paris, France; 5 UMMISCO UMI 209 IRD - UPMC, Bondy, France; 6 State Key Laboratory of Pathogen and Biosecurity, Beijing Institute of Microbiology and Epidemiology, Beijing, China; 7 Ministry of Education Key Laboratory for Biodiversity and Ecological Engineering, College of Life Sciences, Beijing Normal University, Beijing, China; 8 Xi’an Centre for Disease Control and Prevention, Xi’an, Shaanxi, China; 9 Hu County Centre for Disease Control and Prevention, Xi’an, Shaanxi, China; 10 Ministry of Education Key Laboratory for Earth System Modelling, Department of Earth System Science, School of Environment, Tsinghua University, Beijing, China; 11 Finnish Meteorological Institute, Helsinki, Finland; 12 Centre for Ecological and Evolutionary Synthesis (CEES), Department of Biosciences, University of OsloBlindern, Oslo, Norway; Institut Pasteur, FRANCE

## Abstract

Zoonoses are increasingly recognized as an important burden on global public health in the 21^st^ century. High-resolution, long-term field studies are critical for assessing both the baseline and future risk scenarios in a world of rapid changes. We have used a three-decade-long field study on hantavirus, a rodent-borne zoonotic pathogen distributed worldwide, coupled with epidemiological data from an endemic area of China, and show that the shift in the ecological dynamics of Hantaan virus was closely linked to environmental fluctuations at the human-wildlife interface. We reveal that environmental forcing, especially rainfall and resource availability, exert important cascading effects on intra-annual variability in the wildlife reservoir dynamics, leading to epidemics that shift between stable and chaotic regimes. Our models demonstrate that bimodal seasonal epidemics result from a powerful seasonality in transmission, generated from interlocking cycles of agricultural phenology and rodent behavior driven by the rainy seasons.

## Author Summary

Pathogens shared with wildlife cause more than 60% of human infectious diseases. However, there is a scarcity of comprehensive modeling of zoonotic disease dynamics at the wildlife-human interface. Here, we use 30 years of monthly rodent-hantavirus monitoring to show that the complex seasonality in human spillover results from the interplay between an ecological cascade that shapes reservoir infection dynamics and seasonal agricultural cycles that in turn determine human-wildlife contact patterns.

## Introduction

Most emerging infectious diseases are zoonotic, and more than 70% of these originate among wildlife [1]. Zoonotic disease emergence and reemergence has been hypothesized to be driven by environmental and anthropological variability at the human-wildlife interface [2–4]. However, recent reviews of our understanding of the determinants of spillover have shown that a critical knowledge-gap [5] exists as we lack empirically validated models of the ecological interactions between humans, wildlife reservoirs and key environmental drivers [6,7]. In order to untangle the complexity of zoonotic spillover, combined field surveillance and modeling approaches that link the contacts between humans and wildlife with disease dynamics within the wildlife reservoir are essential [8]. However, such comprehensive investigations are still lacking for almost all zoonotic disease systems [9].

Hantaviruses are rodent-borne zoonotic pathogens within the Bunyaviridae family that cause hundreds of thousands of hospitalizations annually on a global scale. Depending on the viral strain in question, which may cause hantavirus pulmonary syndrome (HPS) or hemorrhagic fever with renal syndrome (HFRS), case fatality rates range between 0.5–40% [10]. Hantavirus is responsible for numerous significant zoonotic outbreaks, including the outbreak of HFRS due to Hantaan virus (HTNV) during the Korean War [11], and HPS due to Sin Nombre virus (SNV) in the Four Corners region of the United States in 1993 [12] and more recently in Yosemite National Park, California, in 2012 [13]. Hantaan virus, which is in the clade of hantaviruses that causes HFRS, was first isolated in 1978 [14].

Previous analyses of hantavirus infection dynamics suggest that changes in climate [15–17], environmental condition and/or agricultural activity affect the risk of zoonotic transmission via changes in reservoir dynamics [18,19], exposure risk [20–22], or virus stability in the environment [23–25]. However, HPS/HFRS epidemics do not appear to simply track environmental conditions or rodent dynamics [26,27]. An integrated picture of host-environment interactions and the resulting hantavirus transmission and spillover is far from clear.

**Fig 1:**
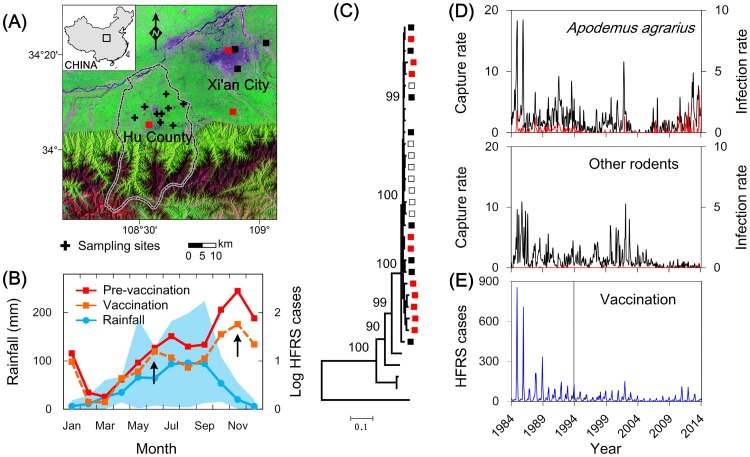
Hantavirus infections and host dynamics in Hu County of central China, 1984–2014. (A) Study area (108° E, 34° N) and sampling sites in Hu County on the Loess Plateau of central China. Crosses indicate rodent sampling sites, squares represent locations of sequences obtained from patients (black) and rodent lungs (red). (B) Mean number of log-transformed reported cases from 1984 to 2014, and the mean monthly rainfall over the same time period, indicating the two peaks of the rainy season (blue line). Monthly number of HFRS cases during the pre-vaccination era (red line): 1984–1993, and vaccination era (orange line): 1994–2014. Blue shaded region gives ±2 standard deviations of rainfall. Arrows indicate bimodal seasonal epidemics. (C) Phylogenetic tree of HTNV in the study area. The black squares represent the sequences obtained from patients, the red squares represent the sequences obtained from rodent lungs (1984–2012), and the hollow squares represent the sequence obtained in this study. (D) The monthly rodent capture rates (black line) and infection rates (red line). All capture rates are expressed as number of rodents caught per 100 trap-nights, infection rates represent number of captured rodent that carry hantavirus per 100 trap-nights. (E) Time series of HFRS cases in Hu County from 1984 to 2014, aggregated monthly.

China has the highest incidence of HFRS worldwide. Our study area in central China, Hu County, is one of the main epidemic areas and serves as a national surveillance site to monitor the HFRS situation. Since 1984, a unique longitudinal field study of hantavirus in rodents with additional epidemiological tracking of human incidence has been conducted in the area (Fig 1A). From 1994 onwards, an attempt has been made to control hantavirus transmission through the targeted routine vaccination of adults aged 16 to 60 yrs. old. Despite these control efforts, the dominant hantavirus (HTNV) continues to infect humans, with dynamics exhibiting clear seasonal and interannual variability as outbreaks invariably coincide with the end of the two rainy seasons (Fig 1B).

In this study, we used a Bayesian state space approach to demonstrate how natural seasonal patterns interact with anthropogenic environmental changes to drive the temporal dynamics of host-virus infection and the consequent risk of HFRS in human populations.

## Results

### Seasonality and dynamics

In Hu County, a total of 9,626 HFRS cases were reported from 1984–2014, with the highest incidence of 0.3% occurring in 1984. During the study, 10,598 rodents were captured in 247,408 trap-nights, with a capture rate of 4 rodents per hundred trap-nights. Of the rodent species captured, striped field mice (*Apodemus agrarius*, mean capture rate of 2.1) was the most frequently captured species, with 48% (5079/10598) of total captures, followed by brown rats (*Rattus norvegicus*, mean capture rate of 0.9), buff-breasted rats (*Rattus flavipectus*, mean capture rate of 0.7), Gansu hamsters (*Cansumys canus*, mean capture rate of 0.3), and house mice (*Mus musculus*, mean capture rate of 0.2) [28].

Hantavirus antigen-positive captures were also found in the rodent species: *A*. *agrarius*, *R*. *norvegicus*, *R*. *flavipectus*, *C*. *canus*, and *M*. *musculus* with positive rates of 6.8% (346/5079), 0.5% (12/2237), 0.2% (4/1702), 0.3% (2/677), and 0.2% (1/493), respectively. Complete S segments of Hantaan virus (HTNV) were obtained from *A*. *agrarius* and HFRS patients from 1984–2012 (Fig 1C, S1 Table) as described both previously and in this study [29,30]. This result indicates that HTNV, carried by *A*. *agrarius*, is primarily associated with the HFRS cases in Hu County. While other rodent species were relatively rarely infected, these sequences were closely clustered with little antigenic variation from sequences obtained from *A*. *agrarius* even over long periods of time (S1 Fig), indicating that these are spillover infection of HTNV. We therefore chose to explore the epidemics of HFRS by considering its dynamics solely within *A*. *agrarius*, using field and laboratory studies.

We found that HFRS epidemics increased after the rainy seasons, even after the mass vaccination program was initiated in 1994, which was associated with a decrease in the mean number of cases (Fig 1B). This suggests powerful seasonality in wildlife-to-human transmission. However, while the incidence indicates a clear intra-annual variability with a strong correspondence to *A*. *agrarius* dynamics (R = 0.80, *P* < 0.05) prior to the vaccination program (Fig 1C–1E), the incidence during the vaccination era testifies to highly erratic outbreaks.

### Ecological cascades

Changes in interannual patterns of epidemics may be linked to changes in potential environment drivers. Environmental variability has cascading effects on wildlife population dynamics [31–33], through breeding success (S2 Fig) and mortality (resource availability and carrying capacity), which may further affect hantavirus dynamics [34]. We propose a mechanistic mathematical model to explore the response of hantavirus dynamics to environmental fluctuations. The model includes the logistic growth of the rodent reservoir [35], where *A*. *agrarius* population dynamics are influenced by environmental factors affecting both birth and death rates, which are in turn determined by amount of rainfall in the breeding season and the carrying capacity of farmland, respectively (see Materials and Methods).

**Fig 2:**
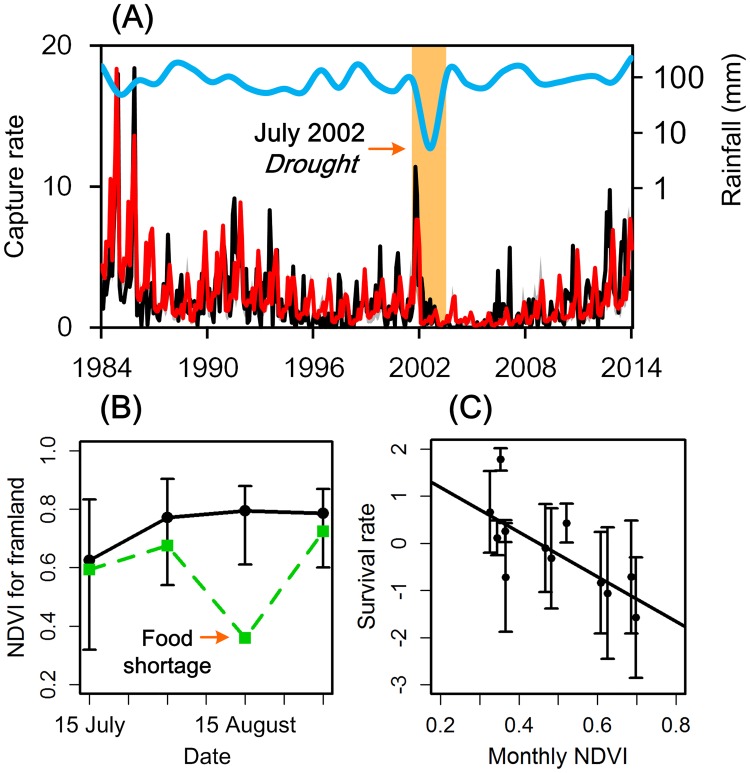
Environmental fluctuations, rodent host dynamics, and hantavirus infections. (A) Rodent population density observed (black line), model-simulated density (red line), and rainfall in July (blue line, unit: mm), 1984–2014. The orange-shaded vertical bars indicate periods of drought. Model-predicted and observed density have a correlation of R = 0.73, *P* < 0.01. (B) Normalized difference vegetation index values for farmland during the drought, from July to August (green dotted line). The black line indicates the average NDVI value of farmland from July to August, 1984–2014. Error bars give ±2 standard deviations. (C) The effect of seasonal changes in NDVI on rodent survival estimated from Eq 2. Error bars show the 95% credible intervals.

The model supports our dynamical hypothesis, and captures the qualitative pattern of rodent population dynamics (Fig 2A). In particular, the model accurately predicts the unusually low abundance between 2002 and 2005. Our analysis reveals that the exceptional 2002 population crash in autumn breeding could be traced back to a significant food shortage, as crops growing after the spring harvest in our study area were affected by extreme drought in 2002. The extent of this catastrophic drought in the area is illustrated by the temperature vegetation dryness index (TVDI) (S3 Fig). In addition, the mean normalized difference vegetation index (NDVI) for farmland in this region was significantly lower during the drought year of 2002, compared with other years (Fig 2B). Significantly, drought may be associated with low breeding rate (S2 Fig), and the resulting food shortage may increase mortality for most rodents (Fig 2C), except the brown rat (*R*. *norvegicus*) which lives in close association with humans and does not rely on farm crops (S4 Fig).

**Fig 3:**
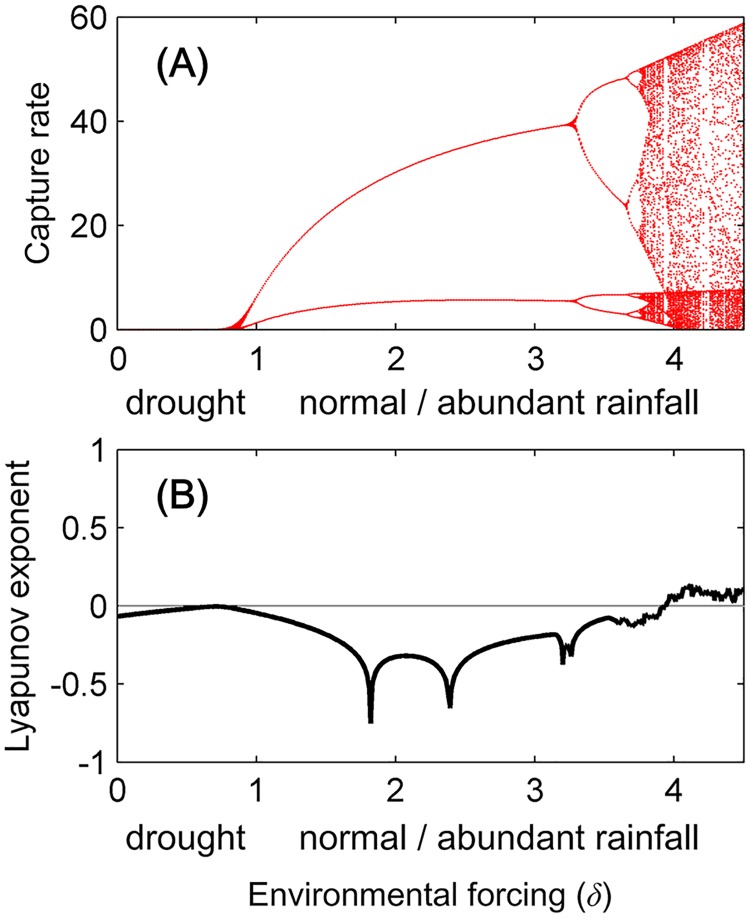
Predicted environmental forcing alters dynamical stability. (A) Bifurcation diagram showing the local minima and maxima of *A*. *agrarius* abundance predicted by the environment-based wildlife dynamic model as a function of the environmental forcing parameter *δ* (Eqs. 12, 13, see Supporting Information). The system heads toward extinction with drought, followed by stable population dynamics with densities that increase as rainfall increases, followed by outbreaks with abundant rainfall and amplitudes that increase with rainfall. Precipitation status (drought/normal/abundant rainfall) is quantified by the corresponding environmental forcing intensity, *δ*, which ranged from 0 to 4.5. (B) Lyapunov exponent versus environmental forcing, predicted by the environment-based wildlife dynamic model. The black line shows the Lyapunov exponent calculated over 100 years with average environmental forcing.

The dynamics of *A*. *agrarius* normally undergoes biennial cycles, which was especially the case in the high population densities of 1984 and 2012. However, these population oscillations collapsed in 2002, initiating a population decline (Fig 1D). We infer that the life cycle of *A*. *agrarius* in our study area is affected by rainfall and resource availability during the breeding season, and our model was therefore constructed to represent these dynamics (S5 Fig). Bifurcation analysis demonstrates that our model produces stable population dynamics (i.e. stable equilibria, in which population numbers remain constant) at low density under low rainfall or drought scenarios, oscillations with 1–2 yrs. periodicity under normal rainfall, and aperiodic and chaotic dynamics (i.e. chaos, in which population numbers change erratically) for strong environmental forcing and abundant rainfall (Fig 3, S6 Fig).

**Fig 4:**
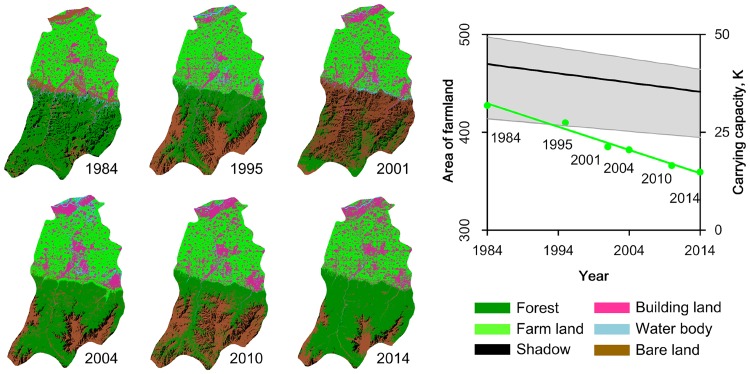
Farmland loss and estimated carrying capacity in Hu County, 1984–2014. Left panel: Land cover maps of Hu County were produced using supervised maximum-likelihood classifications of Landsat Thematic Mapper data. Time series Landsat satellite images, covering the entire city area of Hu County, were used to detect land cover changes, including forest, farmland, building land, bare land, and water. Right panel: Area of farmland (green point) was obtained from remote sensing images, time-varying carrying capacity was estimated from Eq 2. The green line shows the linear regression of farmland area over time, and the black line represents the estimated carrying capacity, and the grey area indicates the 95% credible intervals.

The magnitude of transmission rate varies with time and corresponds to time-varying contacts between susceptible and infected hosts [36]. Thus, environmental changes lead wildlife hosts to a critical density threshold, below which the virus cannot invade (S7 Fig) [34,37]. In addition, a decreased carrying capacity is associated with loss of farmland area over time (Fig 4). This moves hantavirus dynamics into an environmentally forced regime with large fluctuations in infection rate.

### Transmission at the human-wildlife interface

Intervention can explain the discrepancy between rodent dynamics and human infections at interannual timescales. In times of high rodent abundance, the response in number of HFRS cases would be expected to be large. However, the number of HFRS cases was low for 2001 and 2012, despite favorable transmission conditions due to high rodent density (Fig 1). Our results indicate that these episodes were concurrent with a vaccination-induced reduction in human susceptibility. This in turn reduced the number of human hantavirus infections and controlled the susceptible population size, even though the overall human population increased in these decades (see Supporting Information). Most notably, a significant decrease in the number of susceptible individuals was observed after the mass vaccination in 2011 (S8 Fig), and the measures implemented successfully averted further epidemics.

**Fig 5:**
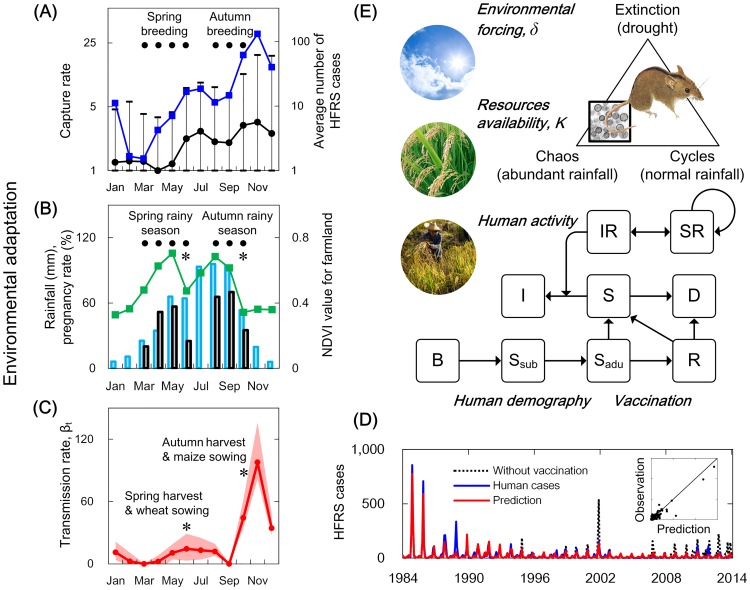
A simplified web of interactions involved in the ecology of hantavirus transmission. (A) Seasonal epidemic and rodent dynamics in Hu County, 1984–2014. *Apodemus agrarius* capture rates (black line) and 95% confidence intervals (whiskers) are shown for 1984 to 2014. The blue line represents the number of HFRS cases. (B) Agricultural phenology and rodent dynamics. Time series of NDVI values of farmland (green line), rainfall (blue bars), and pregnancy rate of rodents (grey bars) [38] are shown. The timing of harvests (*) are denoted, as well as rainy seasons and breeding seasons (black dots). The NDVI value for farmland, which increases gradually during the growth period of crops and suddenly drops during the harvest season, was used to reflect the phenological changes of local crops. (C) Estimated seasonal transmission rate. Shaded regions are the 95% Bayesian credible intervals. Asterisks indicate agricultural activities for the spring harvest and wheat sowing, and autumn harvest and maize sowing. The agricultural activity may increase the risk of human exposure to rodents. (D) Hantavirus infections at the human-wildlife interface. The monthly number of HFRS cases from 1984 to 2014 are shown; the blue line represents observed cases, the red line represents the deterministic prediction from the model (using the susceptible and infected individuals in the first month in 1984 as initial conditions), and the black line represents the predicted outcome of the model without a vaccination campaign from 1994 to 2014. (E) An overview of the system. The series of models link environment to host population dynamics, hantavirus prevalence in rodents, and human demography. The model structures (solid lines) are established according to realistic ecological processes. Susceptible individuals, *S*, were divided into different age groups, births, *B*, deaths, *D*, vaccinated individuals, *V*, immune individuals, *R*, infected individuals, *I*, susceptible rodent hosts, *SR*, and infected rodent hosts, *IR*. The environmental forcing, *δ*, and resource availability, *K*, are incorporated into the model with parameters, which were considered to play a significant role in the fluctuation of rodent populations.

In addition, the number of HFRS cases were clustered on an annual basis during two time periods: June to July, and October to November (Fig 5A). The cases peaked and coincided with two important annual agricultural events, the spring and autumn harvest. Maize is sown in late October for the spring harvest at the end of May, and wheat is sown in June for the autumn harvest at the end of September and October. These agricultural activities coincide with the two rainy seasons (Fig 5B). A second important cycle (shown in Fig 5B) involves the pregnancy rate of *A*. *agrarius* [38], which closely matches the NDVI curve. *Apodemus agrarius*, which tends to live in agricultural fields, initiates its spring breeding season in April–May, and the autumn breeding season starts in August–September [38]. It is interesting to note that the breeding season is closely associated with agricultural activity in Hu County. To summarize, the incidence of HFRS cases peaked during the harvests, when the risk of exposure of farmers to infected rodents in the farmland areas would have increased (Fig 5C, as the average incubation period for HFRS is approximately 3 weeks, ranging from 10 days to 6 weeks [10]). We estimated the seasonal variation in the transmission rate explicitly by applying the discrete-time susceptible-infected-recovered model to 30 yrs. of monthly data from the area, and the fit of the full model accounts for 88% of the variability in the HFRS cases (Fig 5D).

## Discussion

Public health scientists and epidemiologists are increasingly challenged to understand how environmental change and anthropogenic trends affect zoonotic disease dynamics at the wildlife-human interface [39–42]. An effective prevention and control method of zoonotic disease is required, which integrates ecological principles of animal, human, and environmental factors [2,9]. Our study of how shifts in disease ecology can be forced by environmental and anthropogenic processes sheds critical light on zoonotic dynamics and the persistence of disease [2]. We have shown the ecological drivers responsible for the cascading effects of environmental variability on HFRS, using a mechanistic mathematical model integrating longitudinal field surveillance, environmental change and epidemiological data. Once the wildlife and virus dynamics are taken into account, a clear picture emerges of the role of environmental variability in zoonoses [43]. We found support for intra-annual disease cycles driven by seasonal interactions between humans and wildlife, and by an environmentally induced cascade which can switch the dynamics of *A*. *agrarius* abundance between stable and oscillatory [44]. This in turn affects seasonality in HFRS incidence. Our finding adequately explains the complexity and interrelatedness of the environmental, biological, and anthropogenic dimensions of zoonotic pathogen dynamics.

We have provided support for the hypothesis that environmental forcing, rainfall and related vegetation growth, may induce strong chain reactions [45,46] in wildlife dynamics and zoonotic epidemics [47]. Environmental conditions influence survival of the animal reservoir [48,49] and affect transitions between stability, cycles and chaotic dynamics. This is consistent with numerous field studies showing that an increase in resources would allow the rodent host to survive and reproduce [50–52], possibly leading to a higher prevalence of infection [26,53–55] and a higher transmission rate among rodent populations with an older age structure [56] as hantavirus infection is life-long in natural hosts [57]. In turn, this could lead to a greater chance of spillover to humans. Various studies cite HFRS as an example of a zoonotic disease which is linked to climate variability and environmental factors [58–60]. However, general predictions and a supporting model of the effects of environmental change on HFRS dynamics have not yet been empirically tested [27,61,62]. Our results in this study suggest that the proposed mechanism would be valuable in broadening our understanding of human exposure to hantaviruses in general.

Anthropogenic forcing has been linked to disease dynamics and relations between wildlife hosts, humans, and pathogens [63,64]. While prior research has traditionally focused on land-use change and zoonosis emergence [65–67], growing evidence indicates that the expansion of ecotones (transitional areas between adjacent ecological systems) can provide opportunities for pathogen spillover [64,68–70]. Our work provides an improved understanding of the mechanistic processes linking anthropogenic environmental change (for instance, land-use change) and disease dynamics. During the study, farmland loss was found to be associated with host resources and carrying capacity, both of which affect wildlife abundance. In addition, over the past three decades there has been a decline in the abundance of *A*. *agrarius*, coinciding with a long-term trend showing a decrease in the incidence of HFRS.

Our results suggest not only the role of environmental seasonality in shaping population fluctuations [71,72], but also 1) the critical role of human activities, which shape the seasonal dynamics of *A*. *agrarius* by deeply influencing the local rodents’ activity and their life cycles, as well as 2) the role of seasonality in influencing contact between humans and the reservoir host. *Apodemus agrarius* has adapted to thrive in the ecological landscape created by agriculture. This adaptation amplifies seasonality in both transmission and spillover, which alter the spread of infectious diseases [73–75]. The estimated seasonality in the transmission rate shows a bimodal distribution, consistent with the seasonal timing of HFRS cases. Given the distinct roles of wildlife and agricultural activity in transmission, a reasonable explanation for this seasonal pattern is the increase in potential contact between rodents and humans in the dry season due to seasonal agricultural activities. Overall, the combination of both agricultural and seasonal environmental forcing generates a setting in which irregular epidemics arise intrinsically. These findings not only provide evidence for the long-standing hypothesis that environmental change is associated with zoonotic persistence and amplification, but also indicate that the dynamical effects of human-wildlife interactions are dependent on environment-linked processes (Fig 5E).

The HFRS vaccination strategy has been effective and has played an important role in reducing the incidence of HFRS in Hu County [76,77]. Despite this, challenges still remain regarding the prevention and control of HFRS outbreaks. It should be noted that the incidence of HFRS evidently rebounded after 2010, even with high vaccination coverage [19]. This may be attributed to many factors and requires a deeper understanding of the drivers of zoonotic disease risk [78,79]. Taken together with the empirical data on demography and epidemiology, the results suggest that such erratic HFRS epidemics in the study area may have been generated by high amplitude wildlife oscillations interacting with environmental stochasticity and vaccination coverage. All of this demonstrates that wildlife monitoring and modeling may not only help us to retrospectively understand the dynamics of the system, but may also provide advance warning of an outbreak.

Several important limitations of this study should also be acknowledged. First, there is no surveillance data available before 1984, and it is therefore difficult to provide a possible mechanistic explanation for the rodent population peaks in 1984 and 1985. Second, although our rodent surveillance involved constant effort over time, the capture rate was estimated using an unequal number of traps between months. Third, the relationship between environmental variability and the infection rate of wildlife was not considered in the present analysis due to constraints in data availability, and this relationship may have accounted for unexpected outbreaks, e.g. the outbreaks in 1988 and 2011. Future surveillance efforts should include more detailed and frequent sampling of wildlife and hantavirus to improve our knowledge of the association between virus transmission and environmental variability.

Zoonotic diseases significantly impact human health globally, with approximately 1 billion cases and millions of deaths reported each year [63], and are persistent public issues around the world. Our longitudinal survey provides evidence that the key to HFRS epidemic control is critical monitoring of wildlife and the environment, combined with mathematical models to forecast outbreaks and the vaccination of farmers at risk.

## Materials and methods

### Data

The study was located in Hu County (108° E, 34° N) on the Loess Plateau of central China, an area of 1,255 km^2^ and a population of approximately 600,000 people (according to the 2013 Chinese national census). We used the official monthly notification data of HFRS cases from Shaanxi Provincial Center for Disease Control and Prevention and associated demographic data available for Hu County between 1984 and 2014. All HFRS cases were confirmed according to the standard diagnosis set by the Ministry of Health of the People’s Republic of China [80], then confirmed by detecting antibodies against hantavirus in human serum samples. Serum samples were sent to the Shaanxi Centre for Disease Control and Prevention (CDC) for the detection of hantavirus-reactive antibodies. Between 1994 and 2014, a vaccination campaign was conducted in the study area. To assess both the vaccine efficacy and the loss of vaccine efficacy with time elapsed since vaccination, we randomly selected a total of 29,359 people from epidemic and non-epidemic villages in Hu County and monitored them [77]. The health records of each person were investigated, and blood was collected and analyzed by ELISA for the presence of anti-hantavirus IgG specific antibodies.

Starting in 1984, surveillance of the rodent host population density in Hu County has been carried out on a monthly basis (Fig 1B). In each month between 1984 and 2014, rodent trapping was carried out in the fields (farmland or wasteland, 3 km away from villages, which are the habitats for the important rodent reservoirs) in Hu County for three consecutive nights at 9 trapping sites, according to standard protocol from the Chinese Center for Disease Control and Prevention. Snap-traps (medium-sized steel rodent trap, brand name: Golden Cat, Guixi Mousetrap Factory, Jiangxi, China) were baited with peanuts, set each night, and recovered in the morning. At the trapping site, traps were set as 4 parallel lines of 25 traps each and were spaced at 5 m intervals. The trapped rodents were identified to species by species identification experts according to previously described criteria [81]. All rodents were accessioned to the Shaanxi CDC [84HX001-13HX141], and retained as voucher specimens for each species. Lung tissues were removed from the trapped rodents and stored immediately at –196°C, and then transported to the biosafety level-2 (BSL-2) laboratory of Shaanxi CDC for processing. The frozen lungs were sliced with a cryostat (Leica CM1950) and preserved in a refrigerator at –80°C. Tissues and serum specimens for serological or molecular tests were handled during the various laboratory procedures in class II type A2 biosafety cabinets.

The average monthly NDVI, an index of the amount and productivity of vegetation, was derived from satellite data and was generated as follows: NDVI = (NIR—VIS)/(NIR + VIS), where VIS and NIR stand for the spectral reflectance measurements acquired in the visible (red) and near-infrared regions, respectively [82]. NDVI values for farmland were obtained from 9 sampling sites (Fig 1A) during 1984–2014 using AVHRR GIMMS 15-day composite NDVI products [83]. The TVDI, based on an empirical parameterization of the relationship between surface temperature and NDVI, was used in monitoring soil moisture and drought regionally [84]. The TVDI is estimated using the following equation: TVDI = (*T*_s_—*T*_s min_)/(*T*s max—*T*_s min_), where *T*_s_ is the observed land surface temperature at a given pixel, and *T*_s min_ is the minimum surface temperature in the triangle (*T*_s min_ = a_1_ + b_1_(NDVI)). *T*_s max_ is the maximum surface temperature observation for a given NDVI (*T*_s max_ = a_2_ + b_2_(NDVI)). *a*_1_ and *a*_2_ are the intercepts, and *b*_1_ and *b*_2_ are the slopes for the dry and wet edges. The value for TVDI is higher for dry conditions and lower for wet conditions, and varies between 0 and 1. Climatic data, including temperature and rainfall, were obtained from a local meteorological station from 1984–2014.

### Ethics statement

The study’s protocol was conducted according to the guidelines of animal welfare set by the World Organization for Animal Health, and approved by the institutional ethics committee of the Shaanxi CDC (Permit numbers: 2014–2 and 2013–005). The species captured in this study were not protected wildlife and were not included in the China Species Red List, therefore a permit to collect wildlife was not required from an official wildlife/conservation agency.

### Land-use change

During the study, the major crop production in Hu County was spring wheat and autumn maize, occupying most of the local farmland. We used the phenology of local crop production, together with the spectral features of satellite images, to select Landsat images (with a resolution of 30 m) from March to May and September to October in order to extract the areas of cropland in the Hu area in 1984, 1995, 2001, 2004, 2010 and 2014. A support vector machine (SVM) of supervised classification was applied to perform the classification process in ENVI v4.3 [85]. The accuracy assessment of land cover classification using ground truth images by region of interest tools (ROI) indicated a Kappa coefficient of 0.98 on average.

### Detection of hantavirus antigen

Viral antigens in lungs were detected by using direct immuno-fluorescent assay as described previously [86]. Lung tissue samples were cut into 7–8 μm sections on a freezing microtome and fixed in acetone after air drying for at least 30 min. 100μl of FITC-labeled anti-SEOV/L99 or HTNV/76–118 hantavirus nucleoprotein typing monoclonal antibody [87] was pipetted onto each slide. Tissues were incubated at 37°C for 1 hour and washed five times with 0.02 M phosphate buffered saline (PBS). The samples were considered positive when yellow-green fluorescing hantavirus particles were seen under fluorescence microscopy.

### RT–PCR and sequencing

Total RNA was extracted from rodent lung tissue with the TRIzol reagent (Invitrogen, USA) and RNeasy mini kit (Qiagen, Germany), the viral RNAs from the sera of patients were extracted using the QIAamp viral RNA mini kit (Qiagen, Germany) according to the manufacturer’s instructions. cDNAs were synthesized from 5 μg total RNA with the RevertAid first strand cDNA synthesis kit (Fermentas, Canada) in the presence of random hexamers primer according to the manufacturer’s instructions. The partial S segment sequences were obtained by PCR as described previously [29]. The PCR products were gel purified using QIAquick Gel Extraction kit (Qiagen, Hilden, Germany), according to the manufacturer’s instructions. DNA sequencing was performed with the Big Dye Termination Sequencing kit on the ABI-PRISM3730 genetic analyzer (Applied Biosystems, Carlsbad, CA, USA) and sequences (S1 Table) were submitted to GenBank (accession nos. KY357322–KY357327, KY283955–KY283956). For a detailed description of the laboratory methods used, see Ma et al. [29,30].

### Serological assays

Human serum samples were tested for IgG antibodies against hantavirus by ELISA. Briefly, the serum samples were diluted 1:10 in PBS and incubated on a microtiter plate containing hantavirus recombinant nucleoprotein. After incubation at 37°C for 1 hour, the plate was washed six times with PBS containing 0.05% Tween 20 (PBST), then peroxidase-labeled goat anti-human IgG (Millipore, Bedford, MA) at a dilution of 1:10,000 was added. After the incubation and washing steps (described above), tetramethylbenzidine (TMB) and hydrogen peroxide (H_2_O_2_) substrate was added and incubated at 37°C for 10 mins. The reaction was stopped by adding 1 M H_2_SO_4_ and the plates were read on a microplate reader at 450 nm. A net absorbance value of > 0.15 was considered positive.

### Phylogenetic analysis

Neighbor-joining trees of hantaviral S segment sequences were constructed using a GTR + I + Γ4 model in PAUP v4.0b10 [88]. The best-fit phylogenetic model was determined by Modeltest v3.7 [89]. To assess the robustness of the tree topology, a set of 100 pseudoreplicates was generated and used in bootstrap analyses with the maximum likelihood (ML) method implemented in PHYML [90] and the neighbor-joining method implemented in PAUP v4.0b10. A Bayesian phylogenetic tree (10 million generations) was also constructed using MrBayes v3.2 [91]. Trees were highly congruent to those produced above.

### Fitting the TSIR model and ecological fluctuations

We modelled the HFRS epidemics in Hu County (1984–2014) using a discrete-time susceptible-infected-recovered (TSIR) model with age structure [92]. We estimated the seasonality for the HFRS transmission rate by fitting the 30-year-long time series of observed monthly cases using this TSIR model in a Bayesian state-space framework to account for uncertainty in the observation [93]. New infections were drawn from the pool of susceptible individuals, along with information on births, deaths, and vaccinations. As the natural time scale for the disease is ~1 month [94], we used this as the time interval in our model. The number of people susceptible to disease in month *t*+1 is given as *S*_*t*+1_ = *B*_*t*_ + *S*_*t*_*−D*_*t*_−*V*_*t*_−*I*_*t*_ + λ*R*_*t*_. *B*_*t*_ and *D*_*t*_ represent the number of human births and deaths during the time period, respectively. *V* is the number of vaccinated individuals based on medical records, and *R* is the number of immune individuals. *λ* is the proportion of vaccinated people who lost their immunity per month, based on 20 years of surveillance. Susceptible individuals were divided into three different age groups (0–15 yrs., 16–60 yrs., and 61–100 yrs.) according to disease characteristics, and each individual was also kept track of over the study period (Fig 5A). The number of people aged 16–60 yrs. accounted for more than 90% of the total cases in the study area, and as the vaccine was only provided to this group (S9 Fig) we assumed that the highest risk of infection was for this age group. Additional information is given in the Supporting Information.

In the model, the force of infection can be expressed as: *β*_*t*_ (*NR*_*t*_/*N*_*t*_)(*IR*_*t*_), where *N*_*t*_ is the current human population size at time *t*, *IR* and *NR* indicate the number of infected and total rodent hosts, and *β*_*t*_ the month-specific transmission rate from rodents to human beings. The overall human HFRS epidemic dynamics are thus given by:

$$I_{t+1}=\frac{\left(NR_{t}+\tau_{1}\right)}{N_{t}}{\left(IR_{t}+\tau_{2}\right)}^{\alpha }S_{t}\beta_{t}$$ (1)

where *α* allows for the nonlinearities generated by the heterogeneity of the contacts between rodents and humans [95]. The parameter *τ* represents low, random abundances when no animals were caught or no infected animals were caught. Here *β*_*t*_ = *β*_0_(1 + *β*_1_cos(2π*t*)), where *β*_0_ is the average transmission rate and *β*_1_ denotes the amplitude of variation around *β*_0_ [96].

To represent the roles of intrinsic feedbacks from environmental forcing, we proposed an environment-based wildlife dynamic model. The dynamic change of host population can be mathematically represented as:

$$NR_{t+1}=NR_{t}+b_{seas}r_{t}\left(1-\frac{NR_{t}}{K_{t}}\right)NR_{t}-d_{seas}NR_{t}$$ (2)

Hosts grow and die seasonally at rates *b*_*seas*_ and *d*_*seas*_ respectively, which are time-varying parameters influenced by extrinsic drivers. *b*_*seas*_ and *d*_*seas*_ contain both the basic components *b*_*cons*_ and *d*_*cons*_, and an environmental component (influenced by rainfall and the NDVI for farmland). Seasonality has been observed in the birth and pregnancy rates of *A*. *agrarius* [97]. An increase in the number of births during the wet season has been suggested [38], and this was observed in the Chencang district from 1984 to 1987, 10 km away from Hu County, corresponding to a greater percentage of pregnant females during the wet seasons (S2 Fig). Assuming that an increased pregnancy rate is associated with rainfall, we estimated the time-varying seasonal birth rates by monthly rainfall, and set the seasonal birth index, *r*_*t*_, at a value of 0 (non-breeding season) or 1 (breeding season) according to data provided by reference [38]. *K*_*t*_ is the time-varying carrying capacity, determined by the area of farmland (see Supporting Information). To reduce the dimensionality of the model, we ignored sex and age heterogeneity among *A*. *agrarius*.

## Supporting information

S1 Fig Hantaan virus (HTNV) of S segment sequences in China. (A) Map of China, showing the distribution of the S segment of HTNV; the red dot is our study area. (B) Genetic map of HTNV; the HTNV strains present in our study area from 1984 to 2012 are clustered in one branch. (C) Phylogenetic tree of HTNV. The tree was inferred with the Bayesian method using MrBayes. Numerical values at each node indicate posterior probabilities; only values greater than 70% are shown. Sequences obtained from the study area are shown in the red boxes.
(TIF)

S2 Fig Seasonal pregnancy rate of *A*. *agrarius* and rainfall (mm). This data is from the Chencang district, adjacent to Hu County, and was collected between 1984–1987 [38]. The black line is the seasonal pregnancy rate of *A*. *agrarius*, and the blue line represents monthly rainfall (mm), 1984–1987. The pregnancy rate of *A*. *agrarius* was found to be correlated with rainfall (R = 0.60, *P* < 0.01).
(TIF)

S3 Fig Spatial distribution of the TVDI (July 15–30, 2002) in Hu County. The map shows the TVDI, ranging in color from green to red with increasingly dry conditions. The black cross symbols represent rodent sampling locations. The right panel shows the land use of the study area. Land cover maps of Hu County were produced using supervised maximum-likelihood classifications of Landsat Thematic Mapper data. The whole southern part of Hu County is Qin Mountain.
(TIF)

S4 Fig Capture rates of *A*. *agrarius* and *R*. *norvegicus* in Hu County, 1984–2014, and the autocorrelation function (ACF) of *A*. *agrarius* population dynamics. (TIF)

S5 Fig Predictions of the regression model. Observed versus simulated NDVI value for farmland (R^2^ = 0.81). The grey dots indicate actual observations, the red dots indicate simulated data from 1984 to 2003, and the blue dots indicate cross validation for 2004–2014. The predictions are one-step ahead predictions, meaning that the data points at time *t* provide the input for the predictions at time *t* + 1 (see Eq. 11). The model predictions use the same time interval of 1 month as the time series data.
(TIF)

S6 Fig Analysis of the environment-based wildlife dynamic model. Examples of predicted *A*. *agrarius* population densities through time from the independently parameterized model driven by rainfall. Seasonal variation in rainfall, averaged over all years in the time series (blue line), and *A*. *agrarius* dynamics (red line) predicted by the model (Eqs. 12, 13).
(TIF)

S7 Fig Capture rate, infection rate and human cases. (A) Monthly infected rate against the capture rate threshold observed. The vertical line shows the location of the threshold. Capture rate is expressed as number of rodents caught per 100 trap nights, infection rates represent the number of captured rodents that carry hantavirus per 100 trap-nights. (B) Annual dynamics of HFRS outbreaks and *A*. *agrarius*. Both the *A*. *agrarius* population abundance (red bar) and HFRS cases (blue bar) decreased after the drought in 2002 (orange arrow).
(TIF)

S8 Fig Population susceptible to hantavirus over time in Hu County, 1984–2014. (A) The predicted population of Hu County that was susceptible (black line) to hantavirus during the time period 1984–2014. The orange line represents the number of vaccinated individuals based on annual records, and the green line is the total population size. (B) Loss of vaccine efficacy over time. The rate of loss of vaccine efficacy was plotted against the amount of time since the last vaccine dose was received, based on data from our longitudinal studies. The best fit of the logarithmic relationship is shown. These estimates show a logarithmic increase in loss of vaccine efficacy over time since the last vaccine dose was received (orange), consistent with a loss in efficacy of 0.02% per year.
(TIF)

S9 Fig Demography of the vaccine recipients in the study area, 1978–2014. (A) The monthly number of births in Hu County. The numbers are averaged for each year. (B) The monthly number of deaths. (C) The age distribution of HFRS cases and the population demography in Hu County. The vaccine was provided to people aged 16–60 yrs. as people in this age group accounted for more than 90% of the total cases in the study area, and the Pharmacopeia of the People’s Republic of China (2005) specified that vaccines could only be administered to people between 16 and 60 years of age.
(TIF)

S1 Text (DOCX)

S1 Table Sequences used in this study. (DOCX)

S2 Table Variables used in the TSIR model. (DOCX)
