# Supplementary figures and images for: Anthropogenically driven environmental changes shift the ecological dynamics of hemorrhagic fever with renal syndrome
Source: PLoS Pathog. 2017 Jan 31;13(1):e1006198. doi: 10.1371/journal.ppat.1006198 (PMC5302841; doi:10.1371/journal.ppat.1006198)

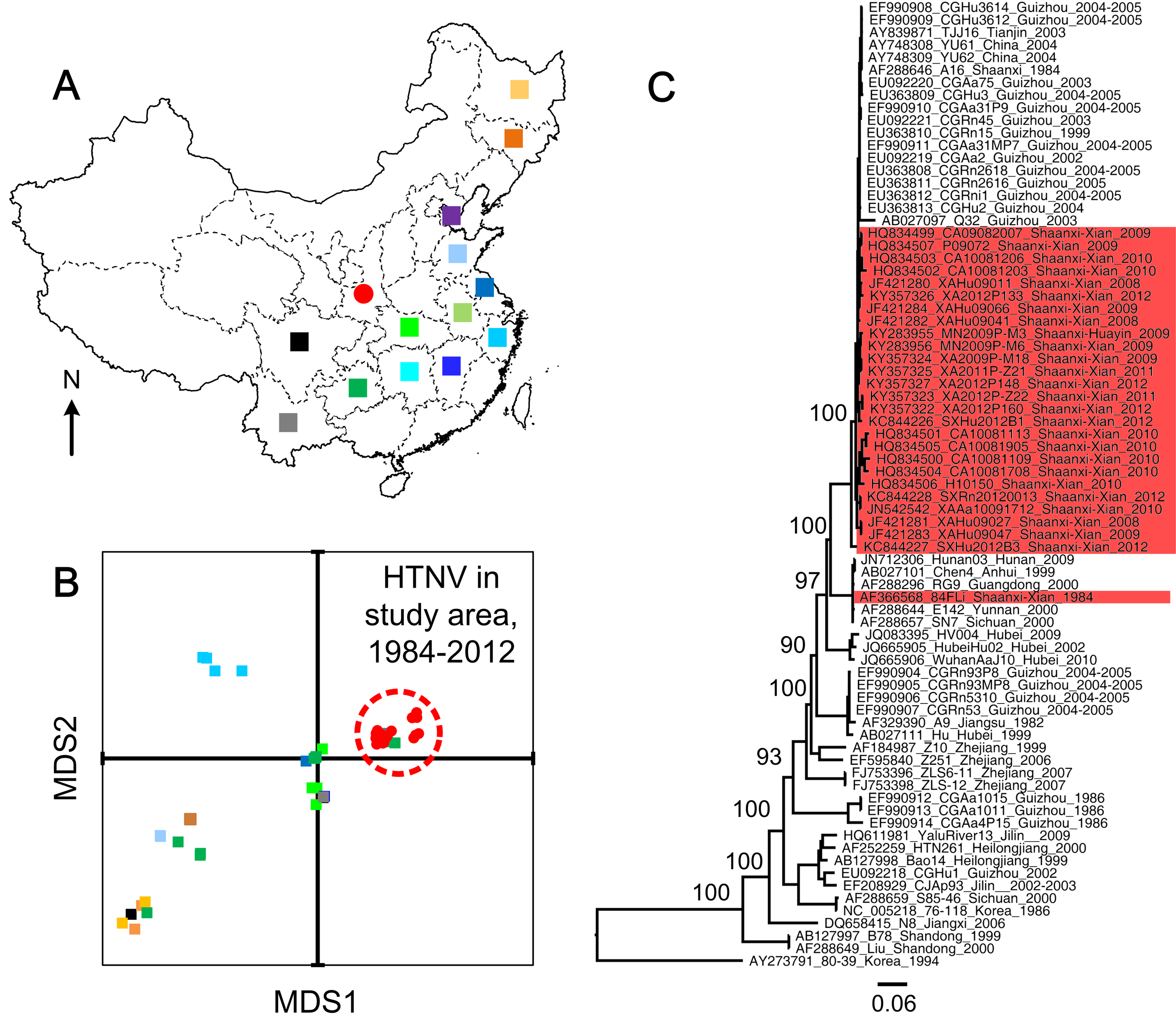

Supplement: S1 Fig — (A) Map of China, showing the distribution of the S segment of HTNV; the red dot is our study area. (B) Genetic map of HTNV; the HTNV strains present in our study area from 1984 to 2012 are clustered in one branch. (C) Phylogenetic tree of HTNV. The tree was inferred with the Bayesian method using MrBayes. Numerical values at each node indicate posterior probabilities; only values greater than 70% are shown. Sequences obtained from the study area are shown in the red boxes. (TIF) [file ppat.1006198.s001.tif]

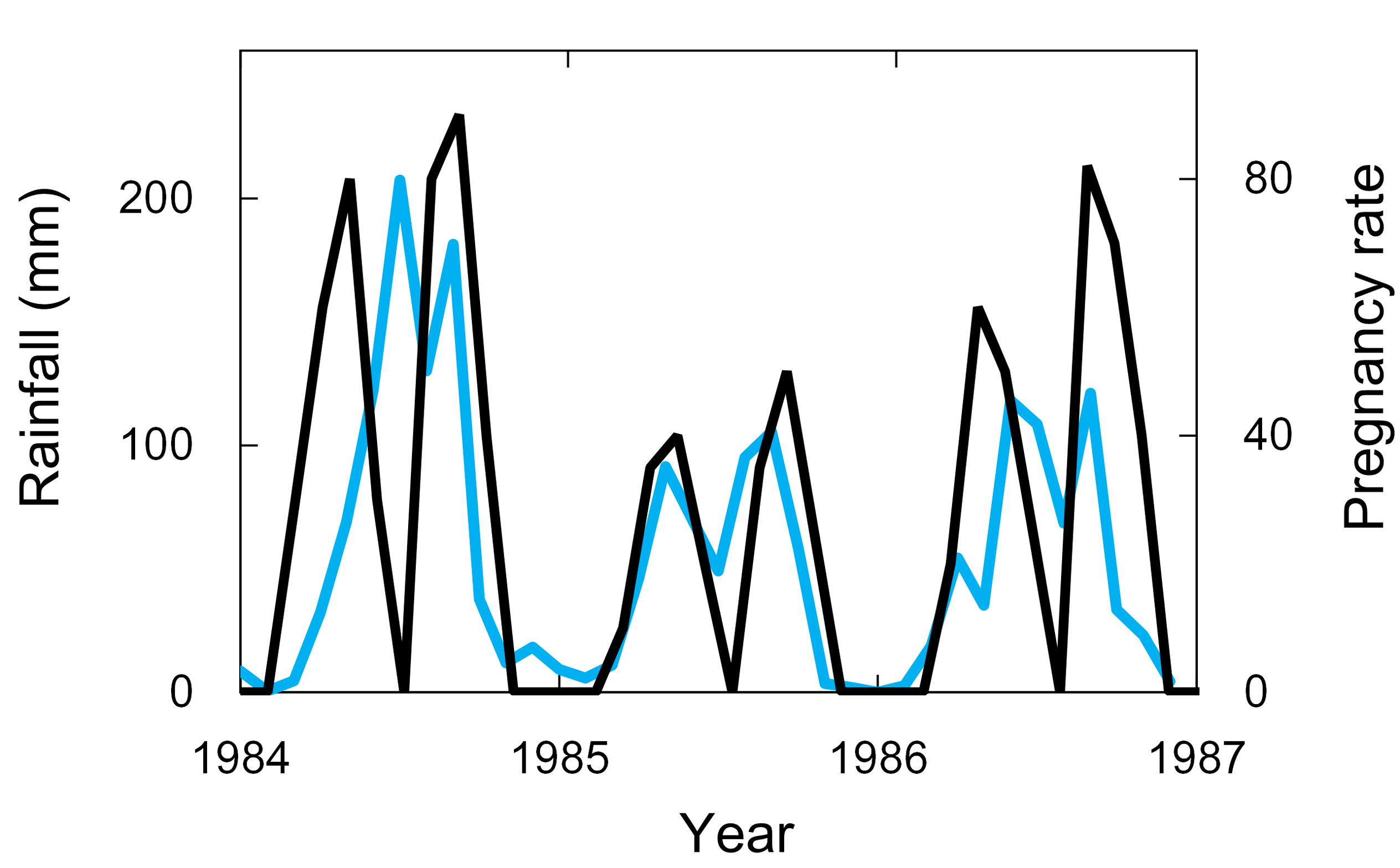

Supplement: S2 Fig — This data is from the Chencang district, adjacent to Hu County, and was collected between 1984–1987 [38]. The black line is the seasonal pregnancy rate of A. agrarius, and the blue line represents monthly rainfall (mm), 1984–1987. The pregnancy rate of A. agrarius was found to be correlated with rainfall (R = 0.60, P < 0.01). (TIF) [file ppat.1006198.s002.tif]

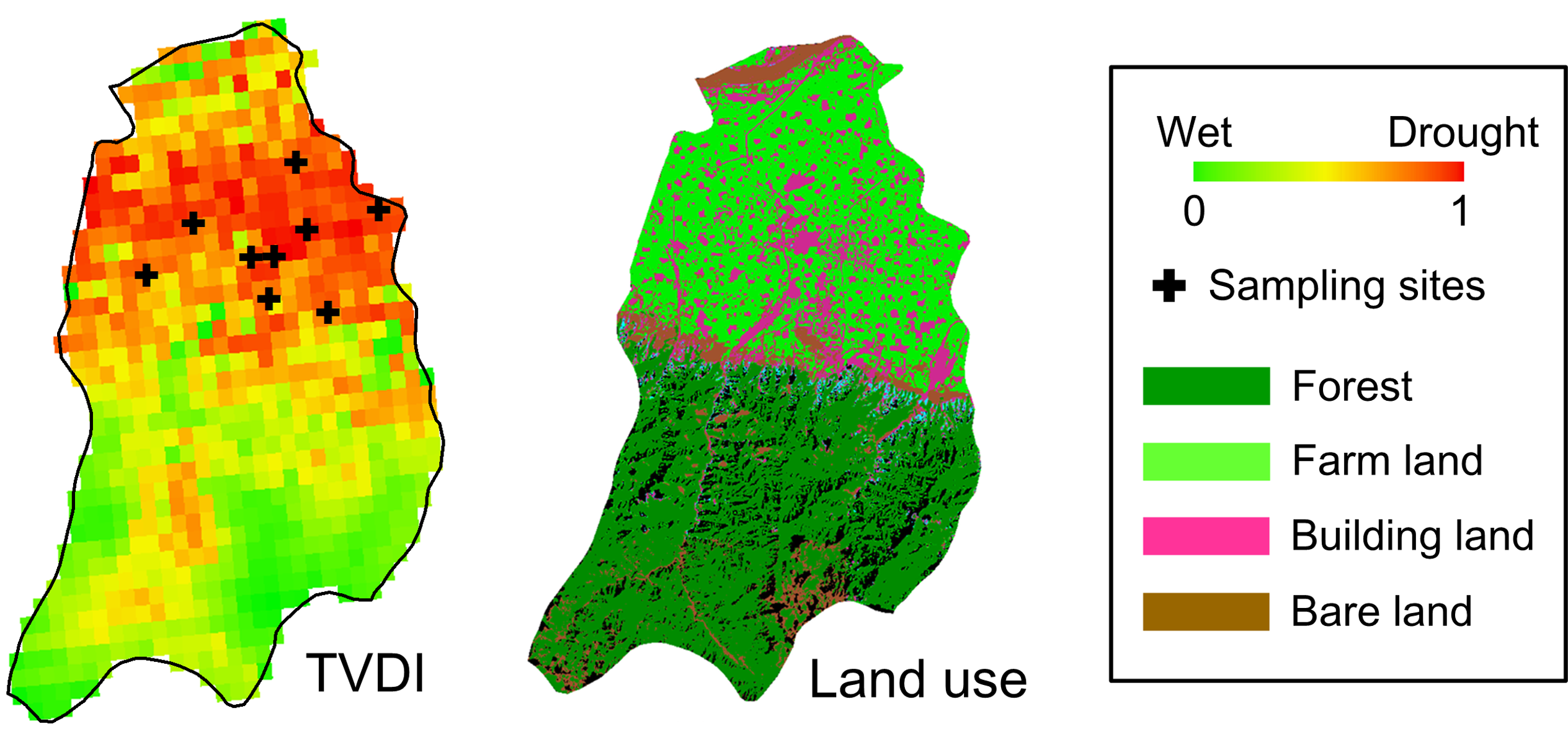

Supplement: S3 Fig — The map shows the TVDI, ranging in color from green to red with increasingly dry conditions. The black cross symbols represent rodent sampling locations. The right panel shows the land use of the study area. Land cover maps of Hu County were produced using supervised maximum-likelihood classifications of Landsat Thematic Mapper data. The whole southern part of Hu County is Qin Mountain. (TIF) [file ppat.1006198.s003.tif]

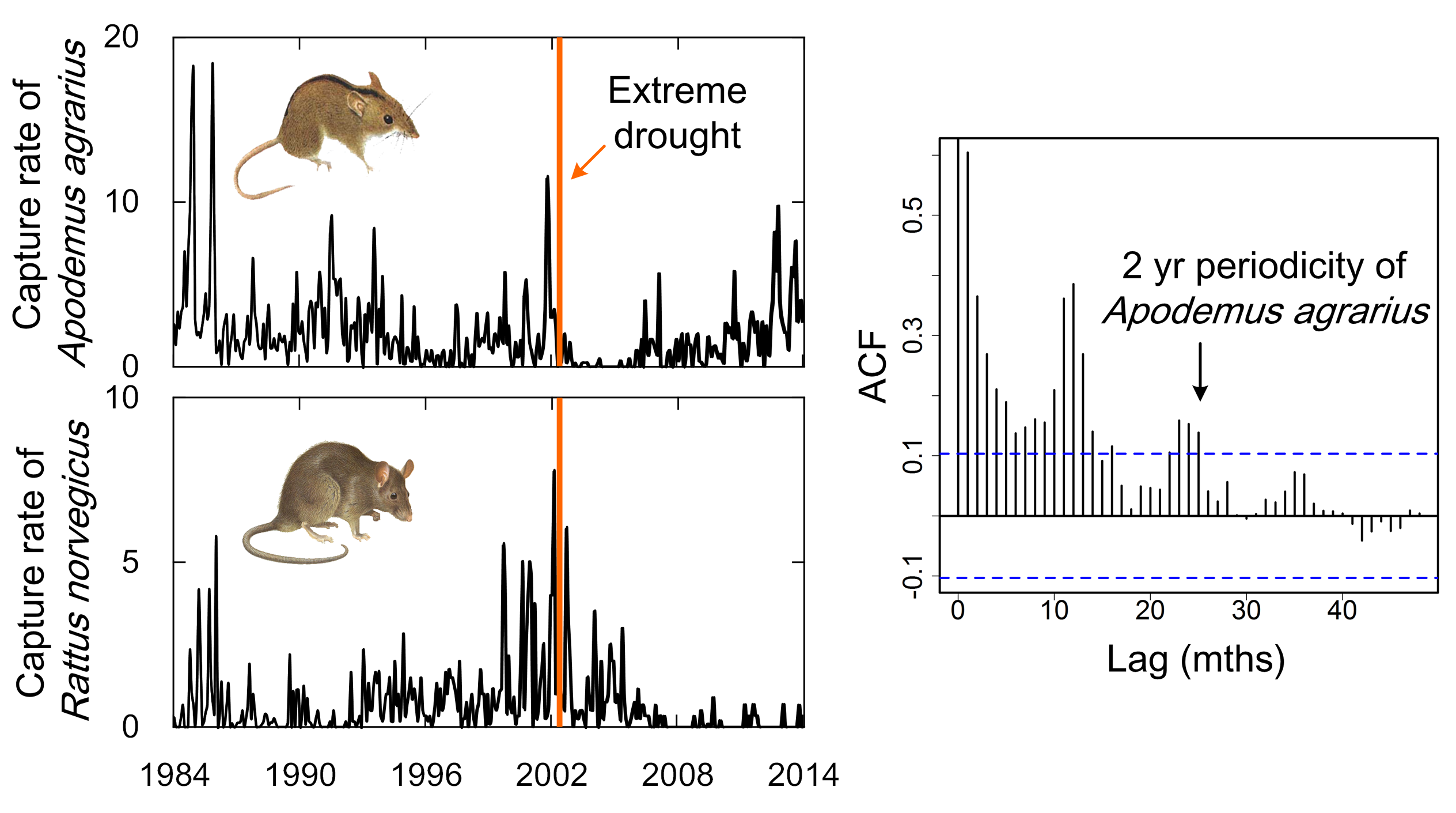

Supplement: S4 Fig — (TIF) [file ppat.1006198.s004.tif]

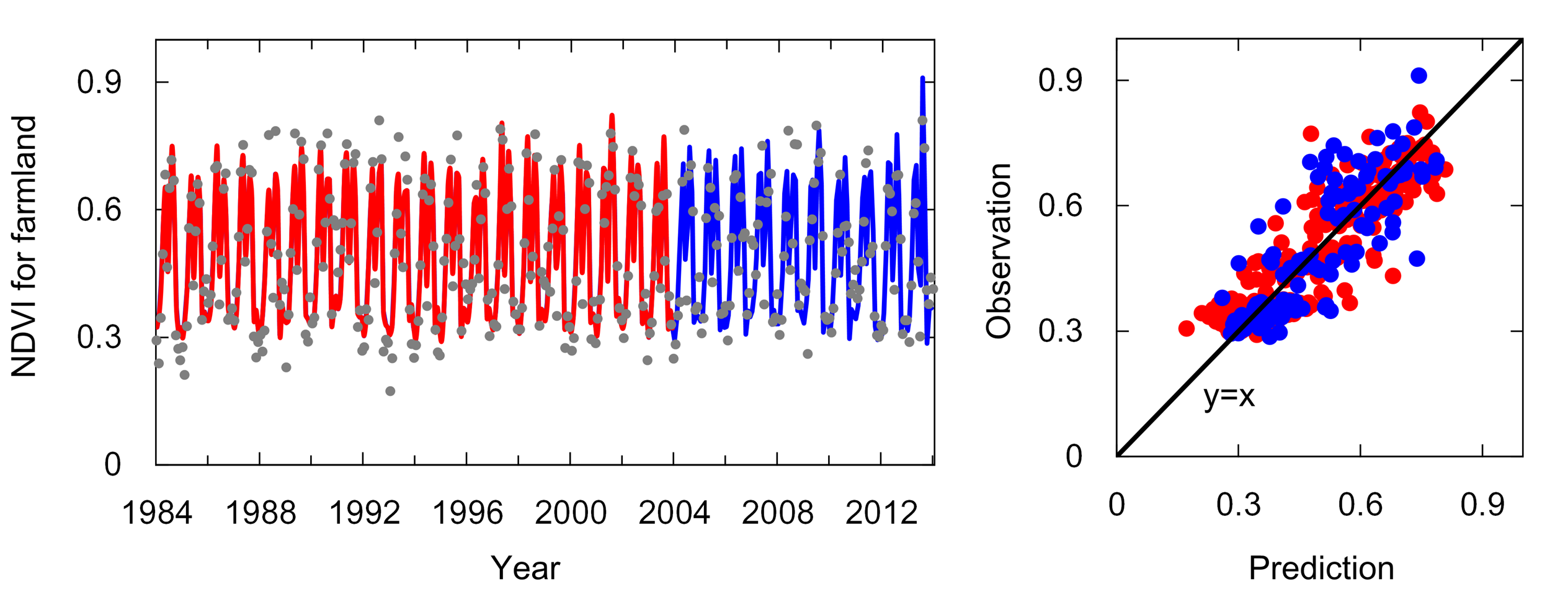

Supplement: S5 Fig — Observed versus simulated NDVI value for farmland (R2 = 0.81). The grey dots indicate actual observations, the red dots indicate simulated data from 1984 to 2003, and the blue dots indicate cross validation for 2004–2014. The predictions are one-step ahead predictions, meaning that the data points at time t provide the input for the predictions at time t + 1 (see Eq. 11). The model predictions use the same time interval of 1 month as the time series data. (TIF) [file ppat.1006198.s005.tif]

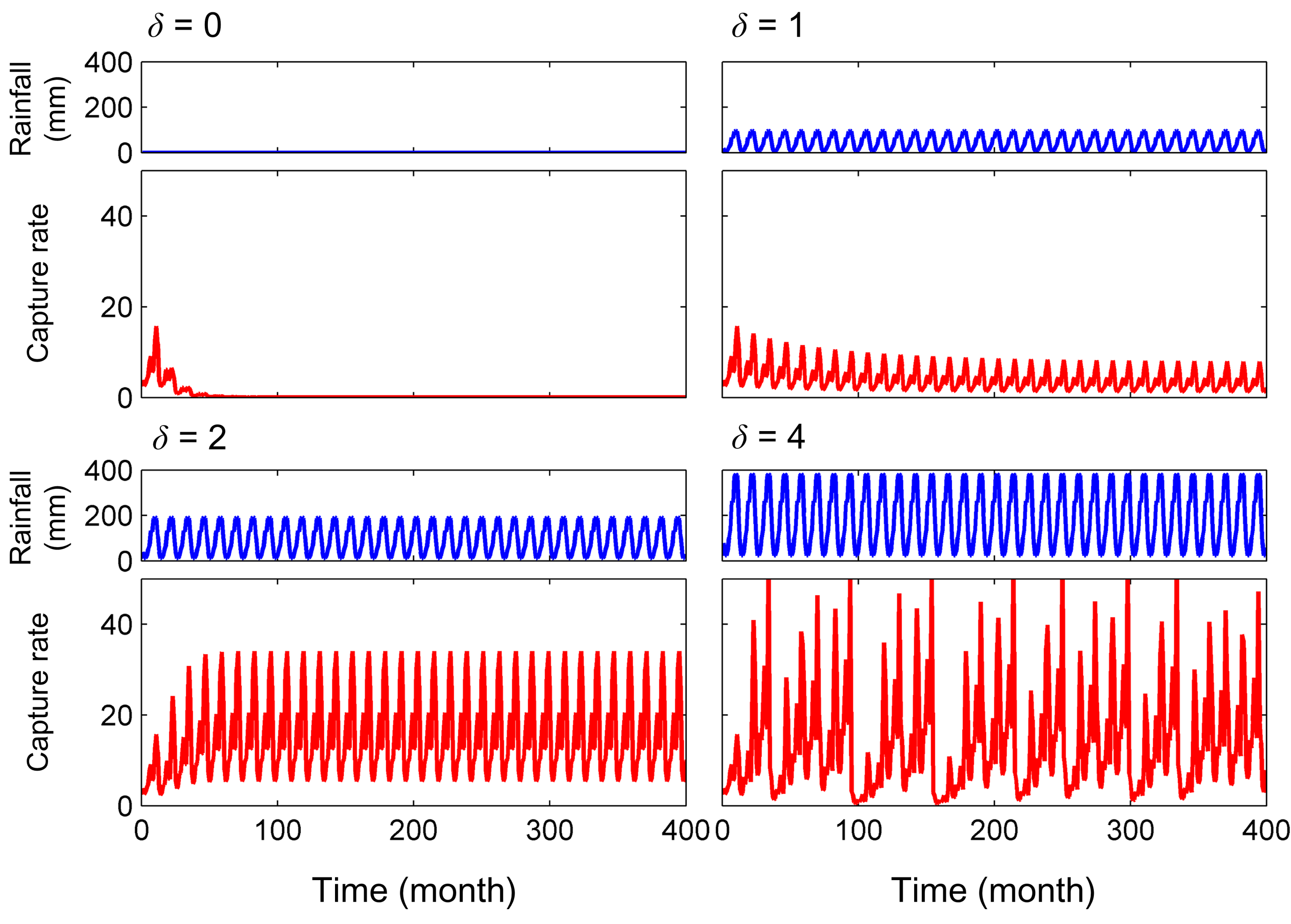

Supplement: S6 Fig — Examples of predicted A. agrarius population densities through time from the independently parameterized model driven by rainfall. Seasonal variation in rainfall, averaged over all years in the time series (blue line), and A. agrarius dynamics (red line) predicted by the model (Eqs. 12, 13). (TIF) [file ppat.1006198.s006.tif]

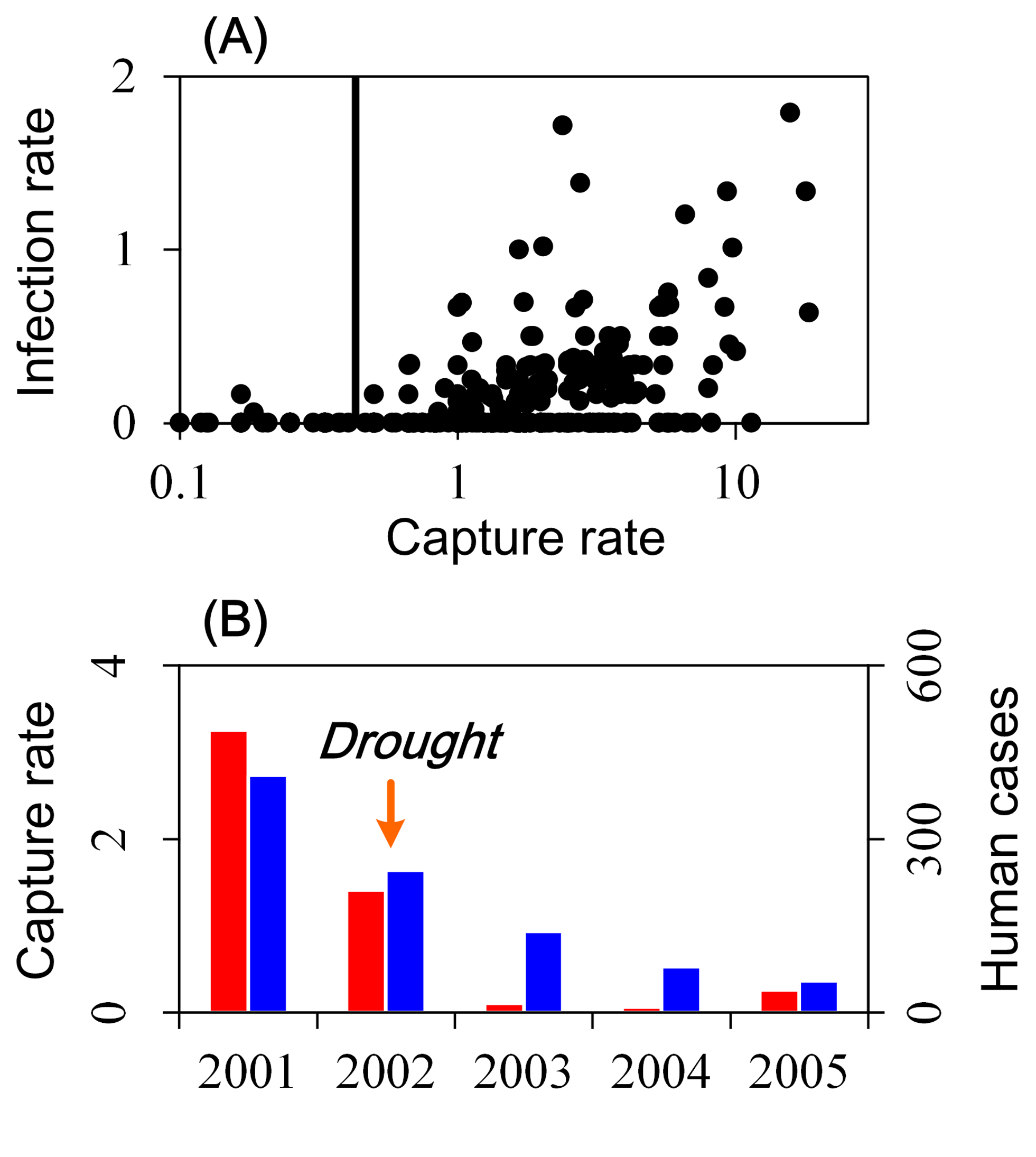

Supplement: S7 Fig — (A) Monthly infected rate against the capture rate threshold observed. The vertical line shows the location of the threshold. Capture rate is expressed as number of rodents caught per 100 trap nights, infection rates represent the number of captured rodents that carry hantavirus per 100 trap-nights. (B) Annual dynamics of HFRS outbreaks and A. agrarius. Both the A. agrarius population abundance (red bar) and HFRS cases (blue bar) decreased after the drought in 2002 (orange arrow). (TIF) [file ppat.1006198.s007.tif]

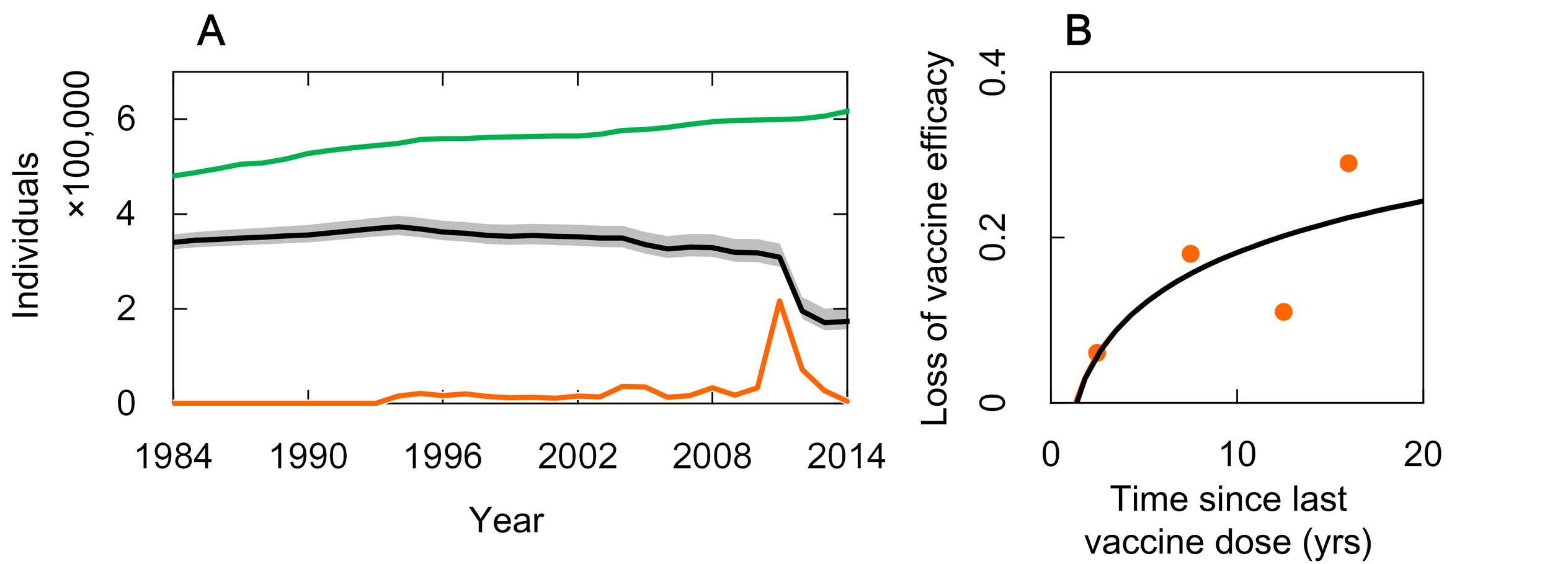

Supplement: S8 Fig — (A) The predicted population of Hu County that was susceptible (black line) to hantavirus during the time period 1984–2014. The orange line represents the number of vaccinated individuals based on annual records, and the green line is the total population size. (B) Loss of vaccine efficacy over time. The rate of loss of vaccine efficacy was plotted against the amount of time since the last vaccine dose was received, based on data from our longitudinal studies. The best fit of the logarithmic relationship is shown. These estimates show a logarithmic increase in loss of vaccine efficacy over time since the last vaccine dose was received (orange), consistent with a loss in efficacy of 0.02% per year. (TIF) [file ppat.1006198.s008.tif]

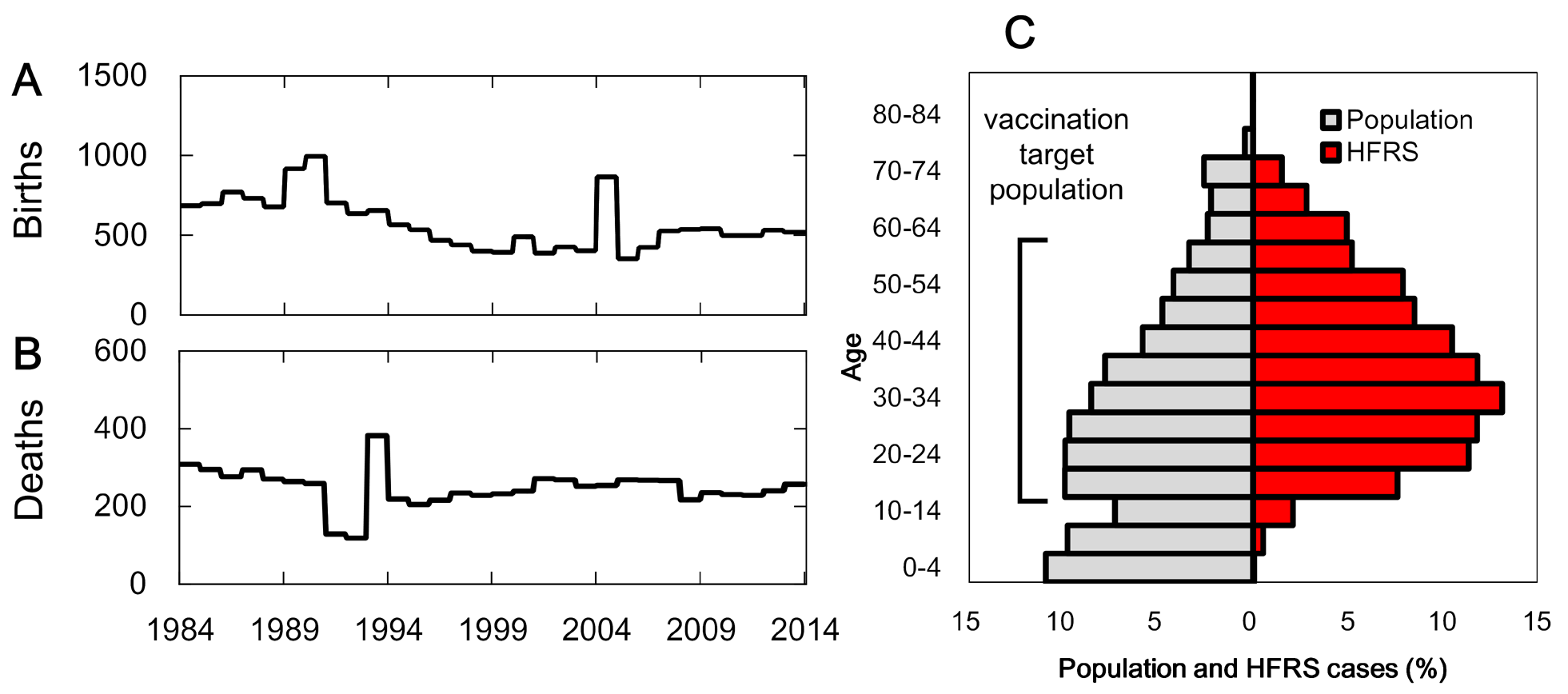

Supplement: S9 Fig — (A) The monthly number of births in Hu County. The numbers are averaged for each year. (B) The monthly number of deaths. (C) The age distribution of HFRS cases and the population demography in Hu County. The vaccine was provided to people aged 16–60 yrs. as people in this age group accounted for more than 90% of the total cases in the study area, and the Pharmacopeia of the People’s Republic of China (2005) specified that vaccines could only be administered to people between 16 and 60 years of age. (TIF) [file ppat.1006198.s009.tif]
