# Supplementary material for: Anthropogenically driven environmental changes shift the ecological dynamics of hemorrhagic fever with renal syndrome
Source: PLoS Pathog. 2017 Jan 31;13(1):e1006198. doi: 10.1371/journal.ppat.1006198 (PMC5302841; doi:10.1371/journal.ppat.1006198)
Supplement: S1 Text — (DOCX) [file ppat.1006198.s010.docx]

**Materials and Methods**

**Fitting the TSIR model and environmental fluctuations**

A discrete time version of the susceptible–infected–recovered model with age structure was developed for describing the HFRS epidemics. The model that incorporated both the human and rodent transmission pathways was fitted on the 30-year-long time series of observed monthly cases in Hu County (1984–2014), using a Bayesian state-space framework to account for uncertainty in the observation [[1](#_ENREF_1),[2](#_ENREF_2)]. New infections were drawn from the pool of susceptible individuals, along with information on births, deaths, and vaccinations. As the natural time scale for the disease is ~1 month [[3](#_ENREF_3)], we used this as the time interval in our model. Thus, the number of susceptibles at time t is given as:

$S_{0-15,t+1}=S_{0-15,t}+B_{t}-S_{16,t}$ [2]

$S_{t+1}=S_{t}+S_{16,t}-S_{61,t}-V_{t}-D_{t}-I_{t}+\lambda R_{t}$ [3]

$R_{t+1}=R_{t}+V_{t}+I_{t}-\lambda R_{t}$ [4]

where *B* and *D* represent the number of births and deaths during the time period, respectively. *V* is the number of vaccinated individuals based on medical records, and *R* is the number of immune individuals. *λ* is the proportion of vaccinated people who lost their immunity per month, based on 20 years of surveillance. Susceptible individuals were divided into three different age groups (0–15 yrs., 16-60 yrs., and 61–100 yrs.) according to disease characteristics, and each individual was also kept track of over the study period. The number of people aged 16–60 yrs. accounted for more than 90% of the total cases in the study area, and as the vaccine was only provided to this group, we assumed that the highest risk of infection was for this age group. Hantavirus infections are thought to cause life-long immunity ^19^, therefore individuals who have recovered should not be susceptible to subsequent infection.

The time series of HFRS cases was specified as a TSIR model, where the force of infection can be expressed as: *β_m_* (*NR_t_*/*N_t_*)(*IR_t_*), where *N_t_* is the current population size at time *t*, *IR* and *NR* indicate the number of infected and total rodent hosts respectively, and *β_m_* indicates the month-specific transmission rate from rodents to humans. The overall human HFRS epidemic dynamics are thus given by:

$I_{t+1}=\frac{\left(NR_{t}+\tau_{1}\right)}{N_{t}}\left(IR_{t}+\tau_{2}\right)^{\alpha}S_{t}\beta_{t}$ [5]

$\beta_{t}=\beta_{0}\left(1+\beta_{1}\cos\left(2\pi t\right)\right)$ [6]

In the above equations, *α* allows for the nonlinearities generated by the heterogeneity of contact between infected rodents and humans [[4](#_ENREF_4)]. The parameter τ_i_ represents low, random abundances when no animals were caught or no infected animals were caught. *β*_0_ is the average transmission rate and *β*_1_ denotes the amplitude of variation surrounding *β*_0_ [[5](#_ENREF_5)]_._

To represent the roles of intrinsic feedbacks from environmental variability, we proposed an environment-based wildlife dynamic model. The dynamic change in the host population can be mathematically represented as:

$NR_{t+1}=NR_{t}+b_{seas}r_{t}\left(1-\frac{NR_{t}}{K_{t}}\right)NR_{t}-d_{seas}NR_{t}$ [7]

$b_{seas}=b_{cons}+r_{t}Rain_{t-2}\sum_{i=1}^{n-1} b_{i}\Delta_{i,t}$ [8]

$d_{seas}=d_{cons}+NDVI_{t-2}\sum_{i=1}^{n-1} d_{i}\Delta_{i,t}$ [9]

Hosts grow and die seasonally at rates *b_seas_* and *d_seas_* respectively, which are time-varying parameters influenced by extrinsic drivers. *b_seas_* and *d_seas_* contain both the basic components *b**_cons_* and *d_cons_*, and the environment influence components *b_i_*Δ*_i_Rain* and *d_i_*Δ*_i_NDVI*, respectively. *b**_i_* and *d_i_* are composed of n = 12 distinct values, one for each month. Δ is a vector of dummy variables with a length of n-1. *r_t_* is the seasonal birth index with values of 0 or 1 referring to a breeding or non-breeding season respectively, estimated from field surveillance. *K_t_* is the time-varying carrying capacity, determined by area of farmland. We assumed that the carrying capacity is an inverse function of the change in farmland area. Thus, the time-specific change in the carrying capacity is given by:

$K_{t+1}=K_{t}\times\Theta\times\frac{P}{Area_{t}}$ [10]

where Θ is the slope of the linear function estimated from the variation of farmland area over the time period 1984–2014. *Area_t_* is the farmland area at time *t*. The term *P* is a scaling constant that reflects the assumption that the carrying capacity may disproportionately decrease over time. To reduce the dimensionality of the model, we ignored sex and age heterogeneity among rodent hosts.

Bayesian modeling with sampling-based methods was used for fitting, and Metropolis-Hastings Markov Chain Monte Carlo (MCMC) was applied for sampling the posterior distributions [[6](#_ENREF_6)]. The Metropolis-Hastings scheme is suitable for sampling from any distribution. The MCMC is implemented using the following steps: First, initial values are chosen for parameters; second, all of these parameters are updated in turn by sampling from conditional distributions using the Metropolis-Hastings algorithm; and third, step 2 is repeated once the Markov chains converge, in order to approximate the marginal posterior distributions. A detailed description of the procedure for Metropolis-Hastings MCMC is provided by Morton and Finkenstädt [[2](#_ENREF_2)]. In the model parameterization, the prior distributions for the parameters were Gaussian, with a mean of 0 and a variance of 10^5^. After a burn-in of 100,000 iterations, we ran the chain for 100,000 iterations sampled every 100th step to avoid autocorrelation. We only present the final results, focusing on the median of posterior distributions and 95% credible intervals.

**Environmental forcing and bifurcation analysis**

We conducted wavelet coherence analysis and phase analysis to quantify the non-stationary relationship between NDVI for farmland and rainfall. The wavelet coherence provides information about where pairs of time series tend to oscillate simultaneously. Phase analysis was then used to characterize the associations between NDVI and rainfall, and to calculate the phase difference of the time lags for the seasonal component. Software for cross-wavelet analysis was developed by Bernard Cazelles, and examples of the application of wavelet analysis and cross-wavelet analysis in ecological and epidemiological time series can be found in the references [[7](#_ENREF_7),[8](#_ENREF_8)].

Based on the results of the cross-wavelet analysis, we proposed a time-series regression model to predict NDVI for farmland (Fig. S6), which included autocorrelation, seasonality, and lagged effect of rainfall. The general model structure, used in the NDVI analysis, was:

$NDVI_{t+1}=\mu_{1}NDVI_{t}+\mu_{2}Rain_{t}+\mu_{3}Rain_{t-1}+\sum_{i=1}^{n-1} \Delta_{i,t}M_{i}+\text{C}$ [11]

where *NDVI_t_* is the monthly normalized difference vegetation index at time *t*, *Rain* denotes rainfall, and C is an intercept term. *μ* is the fixed-effect coefficient for the independent variable, including lagged effect. *M* was composed of n = 12 distinct values, one for each month, denoting month. The model used the same time interval of 1 month as the time series data. We used a cross-validation approach that sampled the first 70% of the dataset for fitting and the last 30% to test the model. Models were fitted using the mgcv package (version 1.7-24) of R version 2.14.

To investigate the environmental variability to the wildlife dynamic model in further detail, we performed a bifurcation analysis using the environmental forcing intensity *δ* as bifurcation parameter (assuming *K* = 40). The model could then be used to study the relative importance of seasonal variation in the environment. Inserting Eq. S11 into Eq. S7 gives:

$NR_{t+1}=NR_{t}+r_{t}\left(b_{cons}+\delta Rain_{t-2}\sum_{i=1}^{n-1} b_{i}\Delta_{i,t}\right)\left(1-\frac{NR_{t}}{K}\right)NR_{t}-d_{seas}NR_{t}$ [12]

$d_{seas}=d_{cons}+\left(\mu_{1}NDVI_{t-3}+\mu_{2}\delta Rain_{t-3}+\mu_{3}\delta Rain_{t-4}+\sum_{i=1}^{n-1} \Delta_{i,t}M_{i}+\text{C}\right)\sum_{i=1}^{n-1} d_{i}\Delta_{i,t}$ [13]

where *Rain_t_* and *NDVI_t_* denote average seasonal rainfall and NDVI value for farmland respectively, from 1984–2014.

The first Lyapunov exponent (LE) was used to quantify the chaotic nature of the deterministic skeleton of the model. It is calculated as the limit of the logarithm of the product of the Jacobian matrix along the model’s trajectory [[9](#_ENREF_9),[10](#_ENREF_10)]:

$\text{LE}=\frac{1}{N\Delta t}\log\left\|\left(\prod_{t=1}^{N\Delta t} \text{J}_{t}\right)\text{U}_{0}\right\|$ [14]

where *N* is the number of data points in the time series, and △*t* is the time interval of the model. J*_t_* is the Jacobian matrix calculated as the partial derivative with respect to *NR* of the wildlife dynamic model (Eq. S12). U_0_ is a unit vector and ||·|| represents the Euclidean vector norm. There are two environmental variables that are important in our study: first, rainfall in the breeding season, and, second, resource availability, which influences *A. agrarius* population dynamics. Thus, we defined the Jacobian matrix that included both partial derivatives with respect to the intrinsic variables, and environmental forcing by rainfall and NDVI for farmland. Then, LE could be used to quantify the effects of environmental forcing, as either amplifying or diminishing, on wildlife dynamics.

We simulated the wildlife dynamic model for 100 years (after withholding the first 30 years as initial transients) for different values of the environmental forcing *δ*. The bifurcation analysis shows periodic *A. agrarius* fluctuations for mild seasonal forcing (0.8<*δ*<3.3), period-doubling in the intermediate range (3.3<*δ*<3.8), and chaotic community dynamics for strong seasonal forcing (*δ*>3.8). Low rainfall or drought (*δ*<0.8) could lead to a low abundance of *A. agrarius*, even a crash in the population, consistent with the observations in 2002 (Fig. S7).

**SI References**

1. Finkenstädt BF, Grenfell BT (2000) Time series modelling of childhood diseases: a dynamical systems approach. J R Stat Soc C 49: 187-205.

2. Morton A, Finkenstädt BF (2005) Discrete time modelling of disease incidence time series by using Markov chain Monte Carlo methods. J R Stat Soc Ser C 54: 575-594.

3. Vaheri A, Strandin T, Hepojoki J, Sironen T, Henttonen H, et al. (2013) Uncovering the mysteries of hantavirus infections. Nat Rev Microbiol 11: 539-550.

4. Liu W, Hethcote HW, Levin SA (1987) Dynamical behavior of epidemiological models with nonlinear incidence rates. J Math Biol 25: 359-380.

5. Dietz K (1976) The incidence of infectious diseases under the influence of seasonal fluctuations. Lect Notes Biomath 11: 1-15.

6. Chib S, Greenberg E (1995) Understanding the metropolis-hastings algorithm. Am Stat 49: 327-335.

7. Cazelles B, Cazelles K, Chavez M (2014) Wavelet analysis in ecology and epidemiology: impact of statistical tests. J R Soc Interface 11: 20130585.

8. Cazelles B, Chavez M, Berteaux D, Ménard F, Vik JO, et al. (2008) Wavelet analysis of ecological time series. Oecologia 156: 287-304.

9. Ott E, Sauer T, Yorke JA (1994) Coping with chaos: Analysis of chaotic data and the exploitation of chaotic systems. New York: John Wiley.

10. Nusse HE, Yorke JA (2012) Dynamics: numerical explorations: accompanying computer program dynamics. New York: Springer.
