# Supplementary material for: Anthropogenically driven environmental changes shift the ecological dynamics of hemorrhagic fever with renal syndrome
Source: PLoS Pathog. 2017 Jan 31;13(1):e1006198. doi: 10.1371/journal.ppat.1006198 (PMC5302841; doi:10.1371/journal.ppat.1006198)
Supplement: S1 Table — (DOCX) [file ppat.1006198.s011.docx]

**S1 Table** Sequences obtained in study area.

| Name | ID | Year |
| --- | --- | --- |
| HQ834499/CA09082007/Shaanxi-Xi'an/2009 | HQ834499 | 2009 |
| HQ834501/CA10081113/Shaanxi-Xi'an/2010 | HQ834501 | 2010 |
| HQ834503/CA10081206/Shaanxi-Xi'an/2010 | HQ834503 | 2010 |
| HQ834500/CA10081109/Shaanxi-Xi'an/2010 | HQ834500 | 2010 |
| HQ834505/CA10081905/Shaanxi-Xi'an/2010 | HQ834505 | 2010 |
| HQ834507/P09072/Shaanxi-Xi'an/2009 | HQ834507 | 2009 |
| HQ834502/CA10081203/Shaanxi-Xi'an/2010 | HQ834502 | 2010 |
| HQ834504/CA10081708/Shaanxi-Xi'an/2010 | HQ834504 | 2010 |
| HQ834506/H10150/Shaanxi-Xi'an/2010 | HQ834506 | 2010 |
| AF288294/LR1/Shaanxi/1979 | AF288294 | 1979 |
| AF366568/84FLi/Shaanxi-Xi'an/1984 | AF366568 | 1984 |
| JF421284/XAHu09066/Shaanxi-Xi'an/2009 | JF421284 | 2009 |
| KC844228/SXRn20120013/Shaanxi-Xi'an/2012 | KC844228 | 2012 |
| JF421280/XAHu09011/Shaanxi-Xi'an/2008 | JF421280 | 2008 |
| JF421282/XAHu09041/Shaanxi-Xi'an/2008 | JF421282 | 2008 |
| KC844226/SXHu2012B1/Shaanxi-Xi'an/2012 | KC844226 | 2012 |
| JF421281/XAHu09027/Shaanxi-Xi'an/2008 | JF421281 | 2008 |
| JF421283/XAHu09047/Shaanxi-Xi'an/2009 | JF421283 | 2009 |
| JN542542/XAAa10091712/Shaanxi-Xi'an/2010 | JN542542 | 2010 |
| KC844227/SXHu2012B3/Shaanxi-Xi'an/2012 | KC844227 | 2012 |
| MN2009P-M3/Shaanxi-Huayin/2009* | KY283955 | 2009 |
| MN2009P-M6/Shaanxi-Xi'an/2009* | KY283956 | 2009 |
| XA2009P-M18/Shaanxi-Xi'an/2009* | KY357324 | 2009 |
| XA2011P-Z21/Shaanxi-Xi'an/2011* | KY357325 | 2011 |
| XA2012P-Z22/Shaanxi-Xi'an/2011* | KY357323 | 2011 |
| XA2012P133/Shaanxi-Xi'an/2012* | KY357326 | 2012 |
| XA2012P148/Shaanxi-Xi'an/2012* | KY357327 | 2012 |
| XA2012P160/Shaanxi-Xi'an/2012* | KY357322 | 2012 |
| AF288646/A16/Shaanxi/1984 | AF288646 | 1984 |

*Reported in this study
