# Supplementary material for: Anthropogenically driven environmental changes shift the ecological dynamics of hemorrhagic fever with renal syndrome
Source: PLoS Pathog. 2017 Jan 31;13(1):e1006198. doi: 10.1371/journal.ppat.1006198 (PMC5302841; doi:10.1371/journal.ppat.1006198)
Supplement: S2 Table — (DOCX) [file ppat.1006198.s012.docx]

**S2 Table** Variables used in the TSIR model

| Parameters | Maximum likelihood estimate | Definition |
| --- | --- | --- |
| *a* | 0.96 (0.75–1.17) | mixing parameter of the contact process |
| *τ*_1_ | 0.73 (0.41–1.05) | low, random abundance of *A. agrarius* |
| *τ*_2_ | 0.42 (0.29–0.54) | low, random abundance of infected *A. agrarius* |
| *β*_0_ | 0.16 (0.02–0.31) | average transmission rate |
| *β*_1_ | 26.71 | amplitude of seasonal variation |
| *b* | 0.09 (0.01–0.17) | basic birth rate of *A. agrarius* |
| *d* | 0.08 (0.06–0.12) | basic death rate of *A. agrarius* |
| *λ* | 0.02 | loss of vaccine-induced immunity |
| *K* | 39.23 (38.40–40.07) | maximum carrying capacity |
| *r* | [0,0,1,1,1,1,0,1,1,1,0,0] | seasonal birth index |
